# Immunotherapy targeting toll-like receptor 2 alleviates neurodegeneration in models of synucleinopathy by modulating α-synuclein transmission and neuroinflammation

**DOI:** 10.1186/s13024-018-0276-2

**Published:** 2018-08-09

**Authors:** Changyoun Kim, Brian Spencer, Edward Rockenstein, Hodaka Yamakado, Michael Mante, Anthony Adame, Jerel Adam Fields, Deborah Masliah, Michiyo Iba, He-Jin Lee, Robert A. Rissman, Seung-Jae Lee, Eliezer Masliah

**Affiliations:** 1 Molecular Neuropathology Section, Laboratory of Neurogenetics, National Institute on Aging, National Institutes of Health, Bethesda, MD 20892 USA; 2 Department Neurosciences, School of Medicine, University of California, La Jolla, San Diego, CA 92093 USA; 3 Department of Pathology, School of Medicine, University of California, La Jolla, San Diego, CA 92093 USA; 4 Department of Biomedical Sciences, Neuroscience Research Institute, and Department of Medicine, Seoul National University College of Medicine, Seoul, 03080 Korea; 5 Department of Anatomy, School of Medicine, Konkuk University, Seoul, 05029 Korea

**Keywords:** Immunotherapy, α-synuclein, Toll-like receptor 2, Transmission, Neuroinflammation, Neurodegeneration, Synucleinopathy, Parkinson’s disease

## Abstract

**Background:**

Synucleinopathies of the aging population are an heterogeneous group of neurological disorders that includes Parkinson’s disease (PD) and dementia with Lewy bodies (DLB) and are characterized by the progressive accumulation of α-synuclein in neuronal and glial cells. Toll-like receptor 2 (TLR2), a pattern recognition immune receptor, has been implicated in the pathogenesis of synucleinopathies because TLR2 is elevated in the brains of patients with PD and TLR2 is a mediator of the neurotoxic and pro-inflammatory effects of extracellular α-synuclein aggregates. Therefore, blocking TLR2 might alleviate α-synuclein pathological and functional effects. For this purpose, herein, we targeted TLR2 using a functional inhibitory antibody (anti-TLR2).

**Methods:**

Two different human α-synuclein overexpressing transgenic mice were used in this study. α-synuclein low expresser mouse (α-syn-tg, under the PDGFβ promoter, D line) was stereotaxically injected with TLR2 overexpressing lentivirus to demonstrate that increment of TLR2 expression triggers neurotoxicity and neuroinflammation. α-synuclein high expresser mouse (α-Syn-tg; under mThy1 promoter, Line 61) was administrated with anti-TLR2 to examine that functional inhibition of TLR2 ameliorates neuropathology and behavioral defect in the synucleinopathy animal model. In vitro α-synuclein transmission live cell monitoring system was used to evaluate the role of TLR2 in α-synuclein cell-to-cell transmission.

**Results:**

We demonstrated that administration of anti-TLR2 alleviated α-synuclein accumulation in neuronal and astroglial cells, neuroinflammation, neurodegeneration, and behavioral deficits in an α-synuclein tg mouse model of PD/DLB. Moreover, in vitro studies with neuronal and astroglial cells showed that the neuroprotective effects of anti-TLR2 antibody were mediated by blocking the neuron-to-neuron and neuron-to-astrocyte α-synuclein transmission which otherwise promotes NFκB dependent pro-inflammatory responses.

**Conclusion:**

This study proposes TLR2 immunotherapy as a novel therapeutic strategy for synucleinopathies of the aging population.

**Electronic supplementary material:**

The online version of this article (10.1186/s13024-018-0276-2) contains supplementary material, which is available to authorized users.

## Background

Following Alzheimer’s Disease (AD), synucleinopathies such as Parkinson’s disease (PD) and dementia with Lewy bodies (DLB) are the second most common group of neurodegenerative disorders of the aging population [1]. Overall, they represent heterogeneous group of neurological conditions, characterized by progressive accumulation of α-synuclein in neuronal and glial cells, selective neuronal degeneration, and neuroinflammatory responses [1–4].

The mechanisms through which the various species of α-synuclein aggregates lead to selective neurodegeneration and neuroinflammation is not completely understood [5, 6]. However, previous studies suggest that α-synuclein oligomers might trigger synaptic dysfunction by interfering with endo-lysosomal transport, mitochondrial function, and calcium dysregulation [5]. Moreover, transmission of α-synuclein aggregates from neuron-to-neuron and neuron-to-glia has been suggested as the underlying mechanism of the neurodegeneration and neuroinflammation in synucleinopathy [1].

We have previously shown that the oligomeric forms of extracellular α-synuclein interact with Toll-like receptor 2 (TLR2) on the surface of neurons and glial cells [7, 8]. While engagement of neuronal TLR2 by extracellular α-synuclein resulted in neurodegeneration by inhibition of autophagy via AKT/mTOR signaling [8], extracellular α-synuclein activated microglia through TLR2 signaling via NFκB and p38 MAPK, thereby resulted in neuro-inflammatory responses with TNFα and IL-6 productions [7]. In addition, recent studies suggested that other receptors such as lymphocyte-activation gene 3 (LAG3) might mediate the pathological effects of α-synuclein transmission [9].

TLR2 belongs to a family of pattern recognition receptor which modulate responses to exogenous pathogens as well as endogenous misfolded proteins released following damage and cellular stress [10]. In the central nervous system, TLR2 is expressed in glial cells and neuronal populations, and recent studies have shown that the levels of TLR2 are elevated in neurodegenerative disorders such as AD and PD [11–14]. Single nucleotide polymorphism in the TLR2 gene has also been associated with PD [15]. Moreover, we have recently shown that inhibition of TLR2 by gene deletion or siRNA-mediated knock down rescues the pathology associated with α-synuclein accumulation in cellular models and transgenic mice [8]. Therefore, TLR2 and downstream signaling have been suggested a new therapeutic target for synucleinopathy [7, 8, 16].

In addition to approaches modulating TLR2 activity by genetic manipulations such as siRNA, more recent studies have also proposed the use of small organic molecules that antagonize TLR2 signaling [17]. While these approaches have some advantages, the main drawback is the low CNS penetration ration and the non-selectivity of small molecules. As an alternative, recent studies have suggested that the immunotherapy blocking α-synuclein [18] and modulating the immune responses might hold some value [19]. For example, neutralizing TLR2 with a monoclonal antibody has been recently shown to ameliorate the pathology in a murine model of AD [14].

We have previously shown that α-synuclein oligomers propagate from neuron to glial cells engaging the TLR2 and promoting inflammation which reduced in the TLR2 knockout background [8], however it is unclear if immunotherapy with TLR2 antibodies might rescue the complex pathology in models of synucleinopathy. In this context, the main objective of this study was to evaluate the therapeutical effects of targeting TLR2 with a functional inhibitory antibody (anti-TLR2) and to better understand the mechanisms action of the immunotherapy by investigating the role of TLR2 dependent pro-inflammatory signaling of extracellular α-synuclein via NFκB. We show that the administration of anti-TLR2 was able to decrease the accumulation of neuronal and astroglial α-synuclein, resulting in reduced neuroinflammation, neurodegeneration, and behavioral deficits in an α-synuclein transgenic mouse model of PD/DLB. Moreover, the anti-TLR2 blocked the neuron-to-neuron and neuron-to-astrocyte α-synuclein transmission and reduced the NFκB dependent pro-inflammatory responses in cell based model. Therefore, TLR2 might be a viable target and TLR2 immunotherapy is a novel therapeutic strategy for synucleinopathies of the aging population.

## Methods

### Antibodies and chemicals

Pam3CSK4 was purchased from InvivoGen (San Diego, CA). The following antibodies were used for western blot analysis, immunostaining analysis, and animal model injection: α-synuclein (Syn-1; BD Bioscience, San Diego, CA), α-synuclein (Syn211), β-actin (Sigma-Aldrich), NeuN, GFAP (GA5), Tyrosine Hydroxylase (Millipore, County Cork, Ireland), Iba1 (Wako, Richmond, VA), TLR2, IL-6, phosphor-NFκB (Abcam, Cambridge, MA), TLR2 (clone T2.5), IgG (eBioscience, San Diego, CA), Active-caspase 3 (R&D systems, Minneapolis, MN), Venus-GFP, and phosphor-p38 MAPK (Cell signaling, Danvers, MA).

### Human specimens, neuropathological evaluation and criteria for diagnosis

Human specimens (8 non-demented controls and 8 PD/DLB cases) were obtained from Alzheimer Disease Research Center/University of California, San Diego. The diagnosis of PD/DLB was based on the initial clinical presentation with dementia followed by parkinsonism and the presence of α-synuclein and ubiquitin positive Lewy bodies in cortical and subcortical regions [20].

### Delivery of lentiviral vectors into mice brain

To determine the role of TLR2 in α-synuclein pathology, we delivered either LV-control or LV-TLR2 into non-tg and α-syn-tg mice expressing human wild-type α-synuclein under the PDGF-β promoter (D line) [21]. Two microliters of either LV-control or LV-TLR2 (2.2 × 10^7^ infection units) was bilaterally stereotaxically injected into the hippocampus (anterior-posterior [AP], − 2.0 mm; medial-lateral [ML], 1.5 mm; and dorsal-ventral [DV], − 1.3 mm). After 5 weeks post injection, mice brains were processed for immunohistochemistry and biochemical analysis. The right hemi-brains were post-fixed in phosphate-buffered 4% PFA at 4 °C for neuropathological analysis, while the left hemi-brains were snap-frozen and stored at − 70 °C for biochemical analysis. All procedures for animal use were approved by the institutional Animal Care and Use Committee at University of California, San Diego under protocol S02221.

### Synucleinopathy mouse model and anti-TLR2 treatment

Transgenic mice overexpressing wild-type human α-synuclein under the mThy1 promotor (α-Syn-tg, Line 61) were used for TLR2 passive immunization analysis since mice develop α-synuclein accumulation in cortical/subcortical regions, neuroinflammation, neurodegeneration, and behavioral deficits [22–24]. Nine-month old mice were injected intraperitoneally (IP) with either control IgG or T2.5 antibodies (5 mg/kg) once a week for 4 weeks. At the end of the study, mice were tested for behavioral defect. Upon termination, the right hemi-brains were post-fixed for neuropathological analysis and the left hemi-brains were stored at − 70 °C for biochemical analysis. All procedures for animal use were approved by the institutional Animal Care and Use Committee at University of California, San Diego under protocol S02221.

### Immunohistochemistry, double-immunolabeling, and neuropathological analysis

The procedures for immunohistochemical, immunofluorescence, double-immunolabeling, and neuropathological analysis have been described elsewhere [25]. Briefly, blind-coded sagittal brain sections were incubated with primary antibodies at 4 °C for overnight. To detect protease K (PK) resistant α-synuclein aggregates, sections were pre-treated with PK (10 μg/ml) for 8 min as previously described [26]. The next day, sections were incubated with either biotinylated-, FITC-conjugated, Texas-red-conjugated secondary antibodies or detected with avidin D-HRP (ABC elite, Vector Laboratories, Burlingame, CA) and with Tyramide Signal Amplification Direct system (PerkinElmer, Waltham, MA), respectively. Sections were imaged by Olympus BX41 microscope. All immunoreactivity levels were determined by optical density analysis using Image Quant 1.43 program (NIH). The cell numbers of GFAP, Iba-1, and NeuN-positive cells were determined per field (230 μm × 184 μm) for each animal based on cell body recognition using Image Quant 1.43 program (NIH).

### Preparation of tissue extract and western blot analysis

The procedures for tissue extraction preparation and western blot analysis have been described elsewhere [8]. Briefly, whole brain homogenates were prepared in the 1% triton-containing lysis buffer, then sonicated. The proteins were separated by electrophoresis and transferred to PVDF membranes using semi-dry Trans-Blot Turbo Transfer System (Bio-Rad, Hercules, CA). Membranes were blocked with Odyssey blocking buffer (LI-COR Biosciences, Lincoln, NE) and probed with primary and followed by fluorescence-tagged secondary antibody. The fluorescent signal detection and densitometric analysis were performed using ODYSSEY CLx (LI-COR Biosciences) and Image Studio (LI-COR Biosciences).

### Behavioral analysis

The evaluation of behavioral defects of synucleinopathy mouse model has been previously described elsewhere [8, 27]. Briefly, to evaluate hyperactivity and anxiety-like behavior of synucleinopathy mouse model, animals were tested in the open field apparatus. Data was collected using a Kinder Smart Frame Cage Rack Station activity monitor system (Kinder Scientific, Poway, CA). Data collection began when an animal was placed in the test chamber. Animals were evaluated for 10 min to determine total activity, latency, and percentage of the time in the periphery vs the center of the box (Thigmotaxis).

### Cell cultures and lentiviral vector infections

The maintenance and differentiation of human SH-SY5Y neuroblastoma, human primary astrocytes, and mouse primary cortical neurons were previously described [7, 8, 28]. Construction and maintenance of V1S and SV2 cells have been described elsewhere [29]. Construction and preparation of lentiviral vectors (LV-control, LV-sh.control, LV-α-Syn, LV-sh.TLR2, and LV-TLR2) have been previously described [8, 30]. To deliver lentiviral vectors, cells were infected with viral vectors at multiplicity of infection of 100 (LV-control and LV-α-Syn) and 50 (LV-sh.control, LV-sh.TLR2, and LV-TLR2).

### In vitro live α-synuclein cell-to-cell transmission monitoring assay

V1S (1.25 × 10^5^) cells were seeded onto trans-well inserts (Corning, Corning, NY) and either SV2 (1.25 × 10^5^) or human primary astrocytes (2.3 × 10^4^) cells were placed in lower compartments on poly-L-lysine-coated glass coverslips. The next day, the trans-well inserts were moved onto the lower compartment to start the co-culture. The lower compartments were harvested at the indicated time point. After incubation, coverslips were fixed with paraformaldehyde (4%) for 30 min in the dark. Fixed SV2 cells were also single immunolabelled against active caspase-3. Fixed human astrocytes were double-immunostained with anti-N-term-venus and anti-IL-6. Coverslips were imaged with a laser scanning confocal microscope Zeiss 800 (Carl Zeiss, Oberkochen, Germany) at 900× magnification. An average 10 fields were analyzed per condition and Image J program was used to determine the pixel intensity. At first images were converted to gray scale then inverted and a mask generated to segment the two cellular compartments. Then the region of interest in the cellular compartment was traced semi-automatically and a threshold was applied followed by estimation of pixel intensity. The diameters of venus punctum were analyzed using Zen program (Carl Zeiss). At least 300 puncta (100 puncta per set, total 3 sets per condition) were analyzed.

### α-Synuclein internalization analysis

dSY5Y neuronal cell, human primary astrocyte, or mouse primary neuron was treated with either LZCM or αSCM (α-synuclein concentration: approximate 1 μg/ml) [7] for indicated hours in the presence of either IgG (5 μg/ml) or T2.5 (5 μg/ml). After an incubation, cells were washed with PBS for 3 times and fixed with 4% PFA. The fixed cells were immunolabelled with antibodies against human α-synuclein, active caspase-3, and/or N-terminus of venus. Coverslips were analyzed with microscope Zeiss 800 (Carl Zeiss) at 900× magnification.

### Quantitative polymerase chain reaction

Extraction of total RNAs and preparation of cDNA from mice brains and cultured cells have been described previously [8, 25]. Quantitative real-time PCR was performed using TaqMan® Fast Advanced Master Mix (Life Technologies) according to manufacturer’s instruction with gene specific primers obtained from Life Technologies, such as TNFα (Mm00443258_m1), IL-1β (Mm00434228_m1 and Hs01555410_m1), IL-6 (Mm00446190_m1 and Hs00174131_m1), CX3CL1 (Hs00171086_m1), CCL5 (Hs00982282_m1) and β-actin (Mm00607939_s1 and Hs03023880_g1). Amplification of DNA products was measured by the StepOnePlus real-time PCR system (Applied Biosystems, Carlsbad, CA). Relative mRNA levels were calculated according to the 2-exp (ΔΔCt) method. All ΔCT values were normalized to β-actin.

### Statistical analysis

InStat (GraphPad Software, San Diego, CA) was used for all statistical analysis. All data were analyzed for statistical significance by using either unpaired *t* test or one-way ANOVA. All data are presented as means ± SEM.

## Results

### TLR2 expression is similarly increased in neuronal and glial cells in α-synuclein transgenic models as is in the brains of patients with PD/DLB

**Fig. 1:**
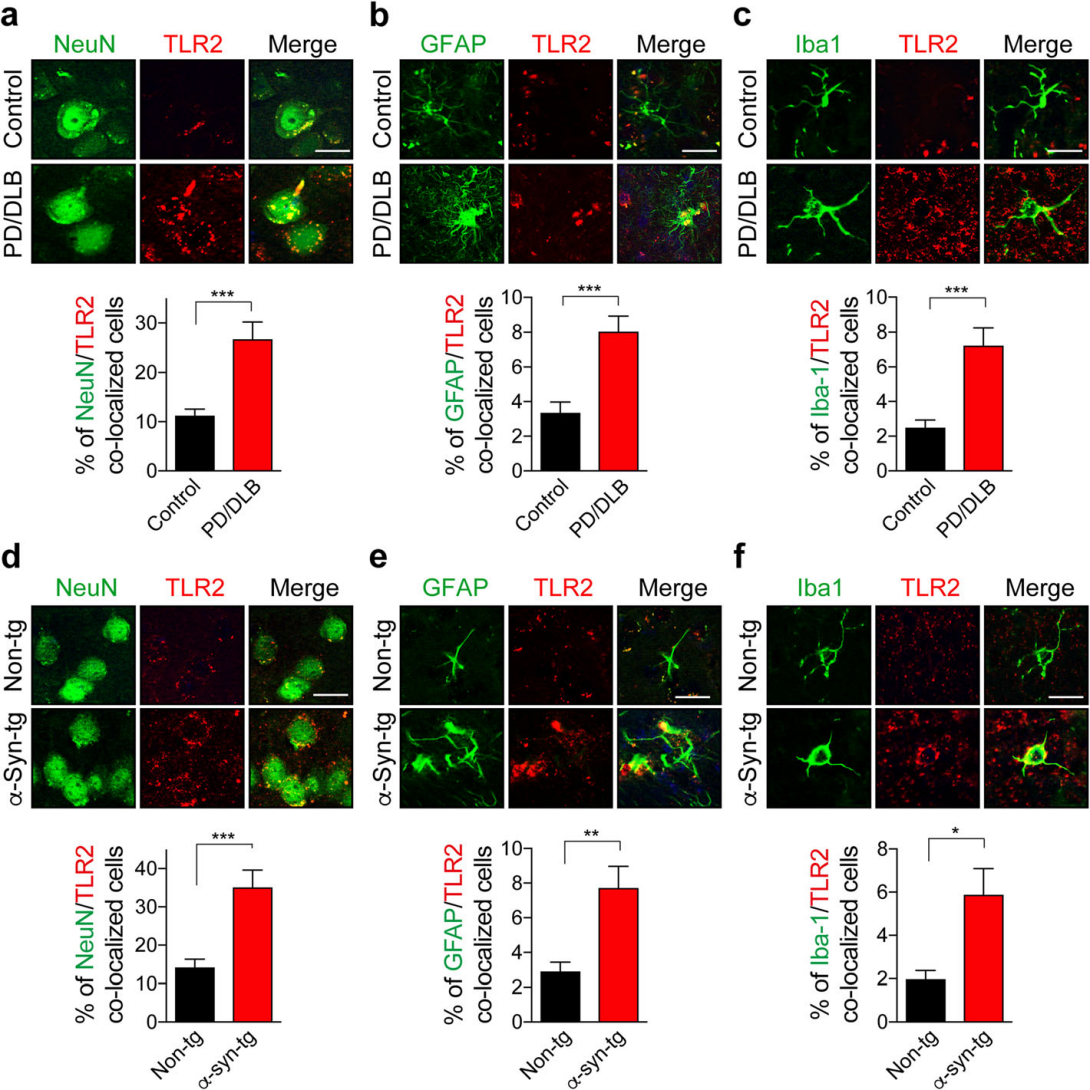
Expression of TLR2 in the neocortex of synucleinopathy patients and an animal model. **a–c** Representative images from double-immunolabeling for TLR2 with cellular markers in the neocortex of normal and PD/DLB patients. The percentages of TLR2-positive neurons (NeuN) (**a**), astrocytes (GFAP) (**b**), and microglia (Iba1) (**c**) in the neocortex (*n* = 8 per group). **d**–**f** Representative images from double-immunolabeling for TLR2 with cellular markers in the neocortex of non-tg and α-Syn-tg (Line 61) mice (9–10 month olds). The percentages of TLR2-positive neuron (NeuN) (**d**), astrocyte (GFAP) (**e**), and microglia (Iba1) (**f**) in the neocortex (*n* = 6 per group). Data are mean ± SEM. *p < 0.05, **p < 0.01, and ***p < 0.001; unpaired t test. Scale bar, 20 μm

We have previously shown that TLR2 might mediate the neurotoxic and pro-inflammatory effects of α-synuclein oligomers [8] and it has been recently reported that levels of TLR2 expression are increased in the brains of patients with synucleinopathy [11–13]. Therefore, antagonizing TLR2 might be able to reverse or prevent the pathological cascades triggered by α-synuclein oligomers. To further validate this possibility, we analyzed the levels of TLR2 in the brains of PD/DLB patients (Fig. 1a-c) and in a transgenic mouse model expressing high levels (3–4 fold) of human α-synuclein under the mThy1 promoter (α-Syn-tg; Line 61) using immunolabeling analysis (Fig. 1d-f) [23]. This α-Syn-tg model (high expresser of α-synuclein) was selected because the mice develop neurodegenerative, neuro-inflammatory, and behavioral deficits similar to patients with PD/DLB [31]. The neocortex of PD/DLB patients and α-Syn tg mice were double-immunolabelled against TLR2 and various cellular markers, such as NeuN (neuron, Fig. 1a and d), GFAP (astrocyte, Fig. 1b and e), and Iba-1 (microglia, Fig. 1c and f). We found that TLR2 expression was increased in pyramidal neurons in the neocortex of patients with PD/DLB and in the α-Syn-tg mice (Fig. 1a and d). Moreover, we found that expression of TLR2 was increased in astrocytes (Fig. 1b and e) and in microglia (Fig. 1c and f) of disease-affected human and mouse brains. Together, these results show comparable increases of TLR2 expression in neurons and glial cells in PD/DLB patients and in the high expresser α-Syn-tg mice and provides rationale to the notion that blocking TLR2 with neutralizing antibodies might be of therapeutic value.

### Overexpression of TLR2 aggravates α-synuclein and related neuropathology in wild-type mice and α-synuclein low expresser transgenic mice

**Fig. 2:**
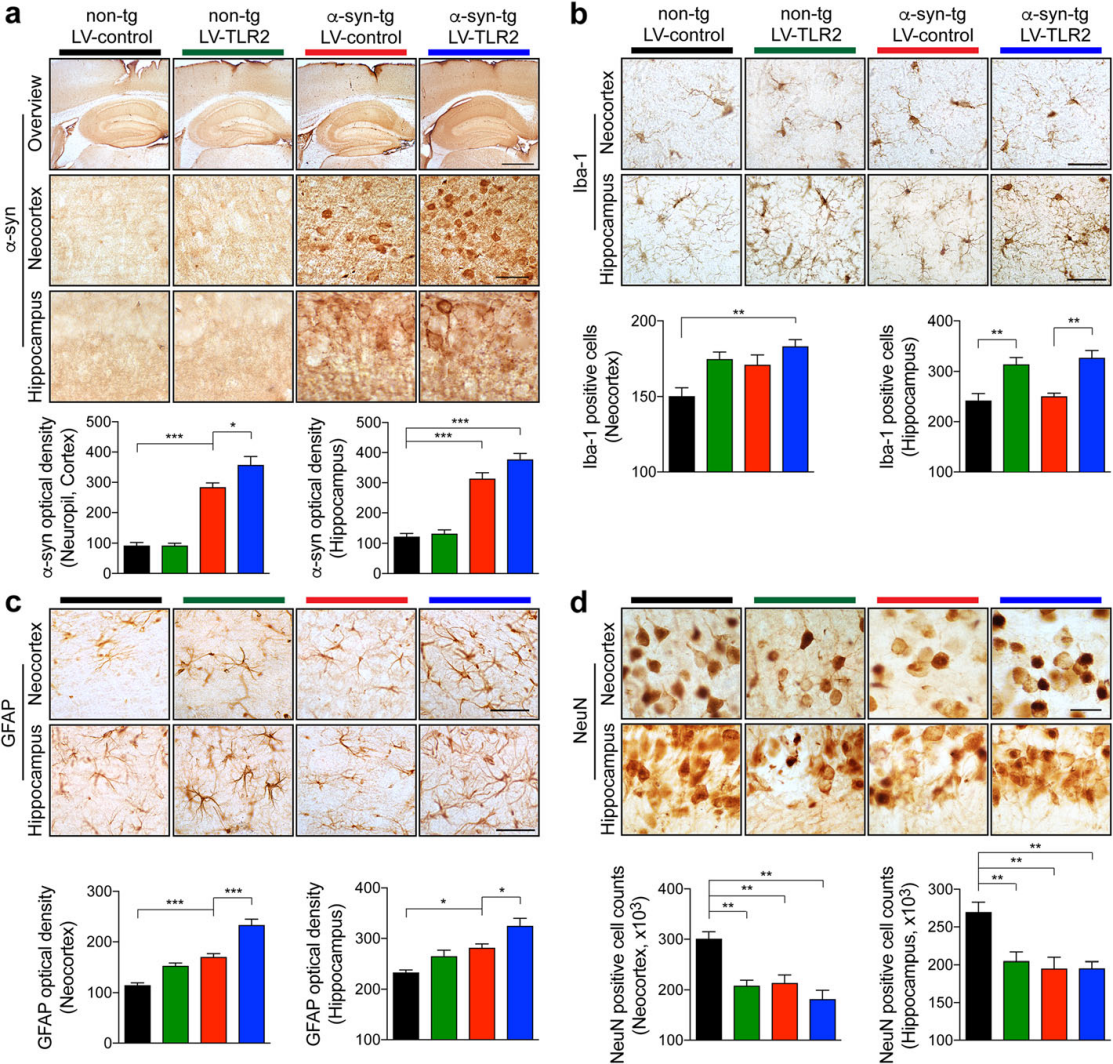
Neuropathology analysis of LV-TLR2-delivered non-tg and α-syn-tg mice. Either LV-control or LV-TLR2 was injected into the hippocampus of non-tg or α-syn-tg mice (D line). **a**–**d** Representative images from immunohistochemical staining of α-synuclein (**a**), Iba-1 (**b**), GFAP (**c**), and NeuN (**d**) in the neocortex and hippocampus of lentiviral vector-delivered mice. The level of α-synuclein (**a**) or GFAP (**c**) was analyzed in the neocortex and hippocampus of the mice by optical density quantification. The number of Iba-1 (**b**) or NeuN (**d**) positive cell was counted in neocortex and hippocampus of the mice. Data are mean ± SEM (n = 6 per group). *p < 0.05, **p < 0.01, and ***p < 0.001; one way ANOVA. Scale bars, 250 μm (low magnification) and 25 μm (high magnification)

To further demonstrate that increasing TLR2 expression triggers neurotoxic and neuro-inflammatory cascades similar to those observed in patients with PD/DLB and in high expresser α-synuclein mouse model line (Fig. 1), we next delivered a TLR2-overexpressing lentiviral vector (LV-TLR2) into the brains of non-tg and an α-synuclein low expresser (1–1.5 fold) transgenic mouse (α-syn-tg, under the PDGFβ promoter, D line) (Fig. 2; Additional file 1: Figure S1) [23]. This α-syn-tg was selected because the lower levels of α-synuclein expression allowing to analyze combinatorial effects with viral vector mediated TLR2 overexpression, moreover we have previously shown that this model mimics aspects of DLB neuropathology and that knocking down TLR2 with shRNA is protective [8]. Either control virus (LV-control) or LV-TLR2 was stereotaxically injected into non-tg and α-syn-tg mice, and then neuropathology was analyzed after a 5-week post injection (Additional file 1: Figure S1a). Delivery of LV-TLR2 increased expression of TLR2 in both α-synuclein-expressing neurons and neighboring glial cells (Additional file 1: Figure S1b and c). Compared to α-syn-tg mice injected with LV-control, the tg mice injected with the LV-TLR2 displayed a dramatic increase in the accumulation of α-synuclein in the neocortex and hippocampus (Fig. 2a). These α-syn-tg mice also display mild neurodegeneration and glial cell activation [32]. Consistent with these findings, compared to the non-tg injected with LV-control, the present study reports mild astrogliosis and microgliosis in the neocortex and hippocampus of the LV-control injected α-syn-tg mouse brains with overexpression of TLR2 considerably increasing neuro-inflammation both in the α-syn-tg mice, but also in non-tg mice (Fig. 2b and c). Interestingly, compared to the non-tg injected with LV-control, the non-tg mice injected with the LV-TLR2 displayed loss of NeuN positive neurons in the hippocampus and neocortex (Fig. 2). As to the LV-control injected α-syn-tg mice, we found loss of neurons in the hippocampus and neocortex when compared to the LV-control injected non-tg mice, however no greater loss of NeuN was observed in the LV-TLR2 injected α-syn-tg mice. (Fig. 2d). We have previously shown [33] that neuron-inflammation and neurodegeneration of this α-syn-tg mice were associated with α-synuclein transfer to astroglial cells, in this context we next investigated if the LV-TLR2 injection will enhance this effect. As expected, no accumulation of α-synuclein was observed in the brain of the lentiviral vector-injected non-tg mice, in contrast LV-control injected α-syn-tg mice displayed discrete accumulation of α-synuclein in glial-like cells and LV-TLR2 injection resulted in a 2.5 fold increase in the brain of α-syn-tg (Additional file 1: Figure S2a). To confirm the identity of these cells, double labeling with anti-GFAP and confocal microscopy was performed. This study confirmed that in the LV-control injected α-syn-tg mice, the α-synuclein in the glial-like cells co-localizes with GFAP and that in tg mice injected with LV-TLR2 there is a considerable increase in α-synuclein/GFAP co-localization (Additional file 1: Figure S2b). Collectively, these results support the concept that increase TLR2 expression might play a role in mediating the neurotoxic and pro-inflammatory effects of α-synuclein. Therefore, it is possible that blocking TLR2 might be of value at ameliorating the pathology associated with α-synuclein accumulation in neurons and glial cells.

### TLR2 passive immunization ameliorates neuropathology and behavioral defect in synucleinopathy mouse model

**Fig. 3:**
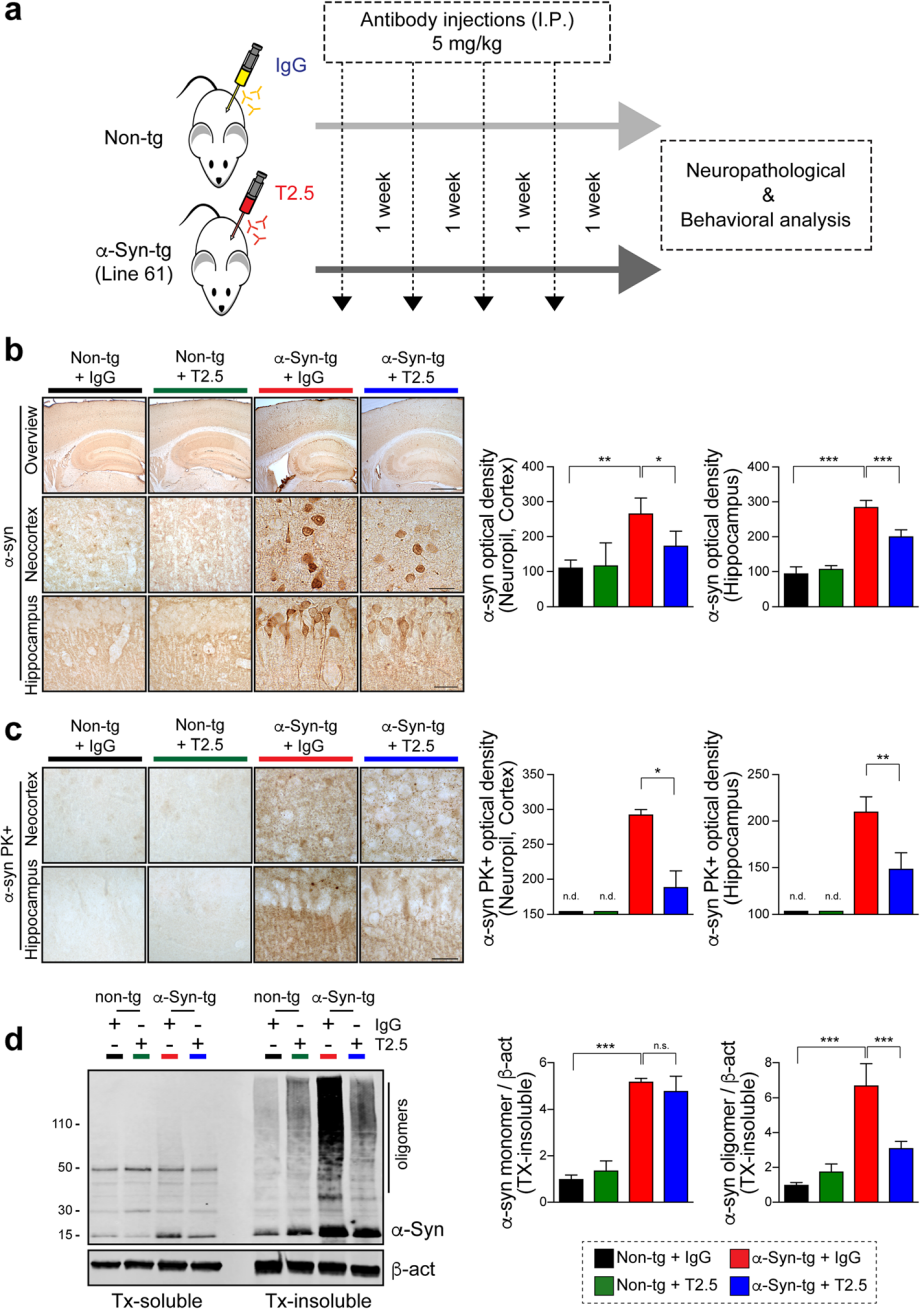
Administration of anti-TLR2 (T2.5) decreases α-synuclein pathology in a synucleinopathy mouse model. **a** Experimental scheme. Non-tg and α-Syn-tg (Line 61) mice were administrated with either IgG (5 mg/kg) or T2.5 (5 mg/kg) weekly for 4 weeks. The levels of α-synuclein pathology, glial cell reactivity, neurodegeneration, and behavioral deficits were analyzed after a 5-weeks post injection. **b** Representative images from immunohistochemical staining of α-synuclein in the neocortex and hippocampus of mice. The level of α-synuclein was analyzed by optical density quantification (n = 6 per group). **c** Representative images from immunohistochemical staining of PK-resistant α-synuclein in the neocortex and hippocampus of mice. The levels of PK-resistant α-synuclein were analyzed by optical density quantification (n = 6 per group). **d** Immunoblot analysis of mice brain lysates. Triton-soluble and -insoluble brain lysates were probed for α-synuclein and β-actin. The levels of triton-insoluble α-synuclein monomer and high molecular weight oligomers were determined by densitometric quantification (*n* = 4 per group). Data are mean ± SEM. *p < 0.05, **p < 0.01, and ***p < 0.001, n.d. not detected; one way ANOVA for (**b**, **d**) and unpaired t test for (**c**). Scale bars, 250 μm (low magnification) and 25 μm (high magnification)

Together with previous studies [8, 34], our current findings support the idea that levels of TLR2 play an important role in promoting the neurodegenerative pathology and deficits in models of synucleinopathy, therefore, we hypothesized that if functional inhibition of TLR2 would reduce overall burden of those pathologies in the model of synucleinopathy. To test this hypothesis, we administrated anti-TLR2 antibody (T2.5, a TLR2 functional blocking antibody) to high expresser α-Syn-tg (Line 61), which mimics neuropathological and functional aspects of PD/DLB including neuroinflammation and increased TLR2 expression (Fig. 1d-f). For this purpose, non-tg or α-Syn tg mice were injected intraperitoneally with either control IgG or T2.5 antibodies (5 mg/kg) weekly for 4 times (Fig. 3a). Approximate 1% of injected antibody may reach the brain according to previous antibody therapy studies [22, 30, 32, 35]. At the end of the study, mice were tested for behavioral effects. Upon termination, the brains were analyzed for biochemical and neuropathological analysis (Fig. 3a).

Compared to IgG treated α-Syn-tg mice, injection of anti-TLR2 significantly reduced the neuronal accumulation of α-synuclein in the neocortex and hippocampus of the α-Syn-tg mice (Fig. 3b). Furthermore, the levels of accumulation of PK-resistant α-synuclein were reduced by anti-TLR2 administration in the neocortex and hippocampus of α-Syn-tg mice, while it was not detected in antibody administrated non-tg mice (Fig. 3c). Likewise, immunoblotting analysis demonstrated that the levels of triton-insoluble high molecular weight α-synuclein oligomers were significantly reduced in the neocortex of α-Syn-tg mouse model after anti-TLR2 administration (Fig. 3d), while triton-soluble and -insoluble α-synuclein monomer was not affected by anti-TLR2 treatment (Fig. 3d).

**Fig. 4:**
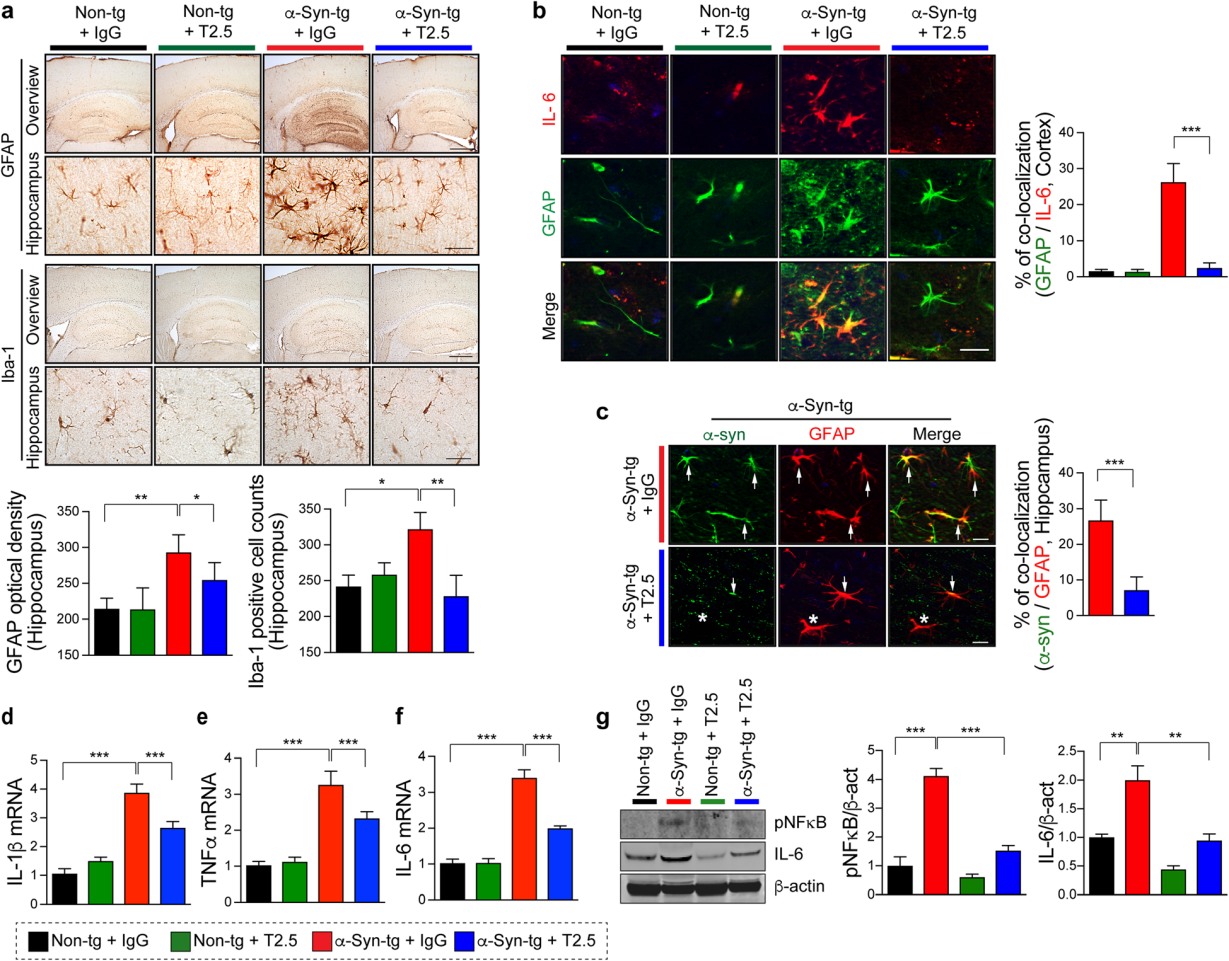
Administration of anti-TLR2 (T2.5) decreases neuroinflammation in synucleinopathy mouse model. Non-tg or α-Syn-tg (Line 61) mice were administrated with either IgG (5 mg/kg) or T2.5 (5 mg/kg) weekly for 4 weeks. **a** Representative images from immunohistochemical staining of GFAP and Iba-1 in the hippocampus of mice. The level of GFAP was analyzed by optical density quantification and the number of Iba-1 positive cell was counted in the hippocampus of mice (n = 6 per group). **b** Representative images from co-localization of GFAP (green) and IL-6 (red) in the antibody-administrated mice. The percentages of GFAP/IL-6 double positive cells were analyzed in the hippocampus of mice. (n = 6 per group). **c** Double immunolabeling analysis for human α-synuclein (green) and GFAP (red) in tg mice. The percentages of α-synuclein and GFAP positive cells were analyzed in the hippocampus of tg mice (n = 6 per group). **d**–**f** Quantitative analysis of the cytokine gene expressions in the cortex of mice. The expressions of IL-1β (**d**), TNFα (**e**), and IL-6 (**f**) were normalized to the levels of β-actin (n = 4 per group). **g** Immunoblot analysis of the whole brain lysates probed for NFκB, IL-6, and β-actin. The levels of NFκB and IL-6 were determined by densitometric quantification (*n* = 3 per group). Data are mean ± SEM. *p < 0.05, **p < 0.01, and ***p < 0.001; one way ANOVA for (**a**, **d**–**g**) and unpaired t test for (**b** and **c**). Scale bars, 250 μm (low magnification) and 25 μm (high magnification)

In addition to the neuronal α-synuclein accumulation in cortical and subcortical brain regions, the higher expresser α-Syn-tg mouse model also displays neuroinflammatory pathology and α-synuclein accumulation in glial cells [25] similar to that of patients with PD/DLB [36]. Compared to non-tg mice, the IgG treated α-Syn-tg mice displayed extensive astrogliosis and microgliosis (Fig. 4a) which was reduced by administration of anti-TLR2 (Fig. 4a). Although microglia, a brain resident immune cell, has been regarded as a major source of cytokine expression, recent studies have shown that astrocyte also could produce inflammatory cytokines and chemokines in response to stimulus [33]. Double immunolabeling analysis against astrocyte marker (GFAP) and IL-6 demonstrated that compared to non-tg mice in the IgG treated α-Syn-tg animals there was an elevation of astroglial IL-6 which was reduced in mice treated with anti-TLR2 (Fig. 4b).

In PD/DLB [36] and tg models [33], previous studies have shown that α-synuclein accumulates not only in neurons but also in glial cells Moreover, we have shown α-synuclein transmits from neuron to glial cells [37], that this is enhanced by TLR2 overexpression (Additional file 1: Figure S2) and that this might result in neuro-inflammation in models of synucleinopathy [33]. Consistent with this possibility, double labeling studies showed that compared to non-tg controls, in IgG treated α-Syn-tg there was considerable co-localization of human-α-synuclein in GFAP-positive astrocytes, in contrast treatment with T2.5 significantly reduced the astroglial accumulation of α-synuclein in the α-Syn-tg (Fig. 4c).

Next, we investigated levels of pro-inflammatory cytokines expression, such as IL-1β, TNFα, and IL-6. We found that compared to non-tg mice, in the IgG treated α-Syn-tg mice there was an increase in the levels of IL-1β, TNFα, and IL-6 expression, however, treatment with the anti-TLR2 antibody normalized levels in the α-Syn-tg compared to non-tg mice (Fig. 4d-f). Consistent with the gene expression findings, immunoblotting analysis demonstrated activation of NFκB and increased IL-6 levels in IgG treated α-Syn-tg mice compared to non-tg controls, while anti-TLR2 administration in the α-Syn-tg showed a reduction in NFκB activation and production of IL-6 comparable to non-tg mice (Fig. 4g).

**Fig. 5:**
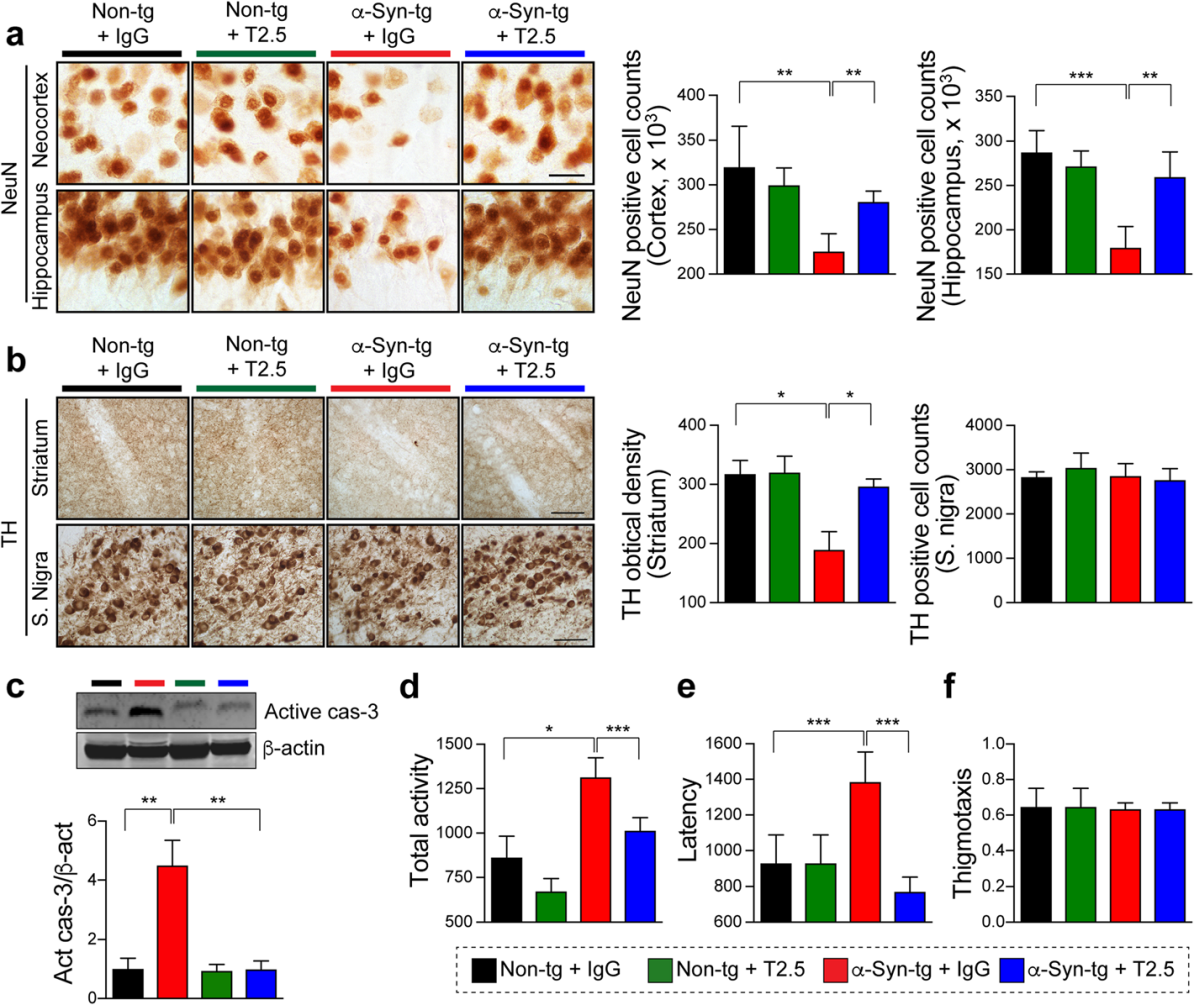
Neuroprotective effect of anti-TLR2 (T2.5) treatment in synucleinopathy mouse model. Non-tg or α-Syn-tg (Line 61) mice were administrated with either IgG (5 mg/kg) or T2.5 (5 mg/kg) weekly for 4 weeks. **a** Representative images from immunohistochemical staining of NeuN in the neocortex and hippocampus of mice. The numbers of NeuN positive cells were counted in the neocortex and hippocampus of mice (n = 6 per group). **b** Representative images from immunohistochemical staining of Thyroxine hydroxylase (TH) in the antibody-administrated mice. The level of TH was analyzed by optical density quantification and the numbers of TH positive cell were counted in the striatum and substantia nigra of mice, respectively (n = 6 per group). **c** Immunoblotting analysis of whole-brain lysates. The lysates were probed for active form of casepase-3 and β-actin. The level of active caspase 3 was determined by densitometric quantification (n = 3 per group). **d**–**f** Behavioral analysis of the mice. The total activity (**d**), latency (**e**), and thigmotaxis (**f**) were analyzed by open field test (n = 6 per group). Data are mean ± SEM. *p < 0.05, **p < 0.01, and ***p < 0.001; one way ANOVA. Scale bar, 25 μm

Following this step, we analyzed if anti-TLR2 administration had effects on the neurodegenerative pathology in the α-Syn-tg mice (Fig. 5). We have previously shown that these mice develop loss of neurons in the deeper layers of the neocortex and CA3 of the hippocampus [38] and loss of Tyrosine hydroxylase (TH)-fibers in the striatum [24]. Compared to non-tg mice, IgG treated α-Syn-tg mice displayed loss of neurons in the neocortex and hippocampus that was prevented by the treatment with anti-TLR2 (Fig. 5a). Likewise, compared to non-tg mice, IgG treated α-Syn-tg mice displayed loss of TH-positive fibers in the striatum and anti-TLR2 administration significantly ameliorated the loss of TH-positive fibers in the α-Syn-tg (Fig. 5b). Consistent with previous studies, no difference was observed in the numbers of TH positive neurons in the substantia nigra of antibody-administrated non-tg and α-Syn-tg mice (Fig. 5b). In agreement with the immunocytochemical evaluation of neurodegeneration, immunoblotting analysis of brain homogenates also demonstrated that compared to non-tg mice, the active form of caspase-3 was increased in the IgG treated α-Syn-tg, however anti-TLR2 administration normalized levels in the α-Syn-tg to those observed in non-tg mice (Fig. 5c).

To determine if the reduction of neuropathology had functional consequences, we performed open filed test to evaluated the total activity and anxiety-like behavior of α-Syn-tg mouse model (Fig. 5d-f). We have previously shown that neurodegeneration and neuro-inflammation in these mice is associated with hyper-activity [24]. Compared to non-tg mice the IgG treated α-Syn-tg showed a significant increment of the total activity (Fig. 5d). Treatment of α-Syn-tg with anti-TLR2 clearly normalized the total activity to levels comparable to the non-tg mice (Fig. 5d). Similarly, compared to non-tg mice, the latency level was increased in IgG treated α-Syn-tg, while anti-TLR2 administration significantly normalized this behavior (Fig. 5e). Finally, the levels of thigmotaxis (a marker of anxiety) were not altered by α-synuclein overexpression as well as antibody administration in the mice (Fig. 5f).

Collectively, these results suggest that targeting TLR2 by immunotherapeutic approach decreased accumulation of neurotoxic α-synuclein aggregates and neuroinflammation, thereby ameliorating neurodegeneration and behavioral defects in an animal model of synucleinopathy.

### Neutralizing TLR2 inhibits abnormal accumulation of neurotoxic α-synuclein in neuron

**Fig. 6:**
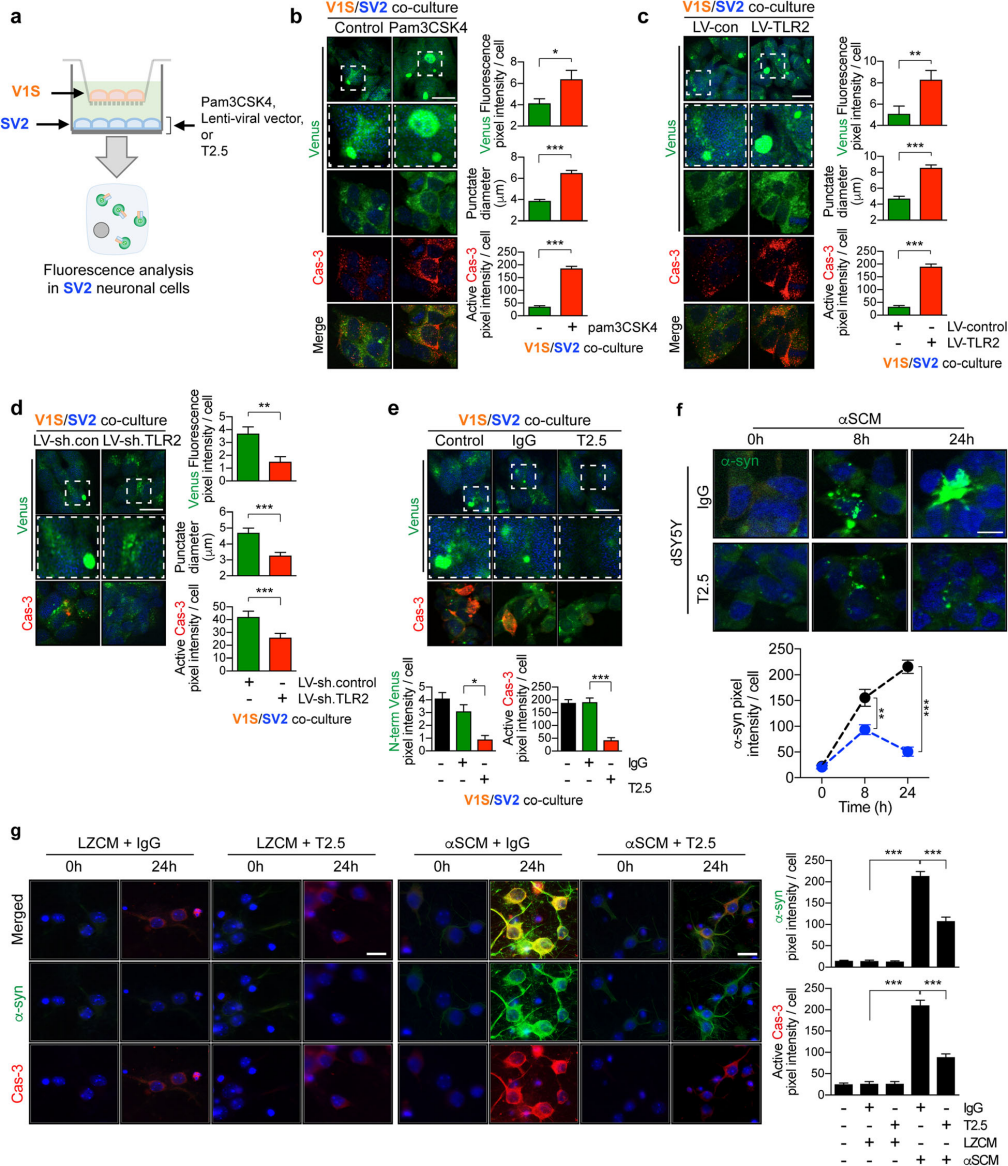
TLR2 mediates neurotoxic neuron-to-neuron α-synuclein transmission. **a** Overview diagram. Donor neuronal cells (V1S), expressing α-synuclein-conjugated with N-terminus of venus were plated in trans-well insert and the recipient neuronal cells (SV2), expressing α-synuclein conjugated with C-terminus of venus were seeded onto cover slips in the bottom well. Only SV2 cells were treated with pam3CSK4 (10 μg/ml), lentiviral vectors, or antibodies. Images were taken from SV2 cells after a 3-days co-culture. **b**–**e** Representative confocal images for BiFC fluorescence and caspase-3 activity in SV2 cells. Middle panels are enlargements of cropped regions outlined with dashed lines from upper panels. Lower panels are double-immunolabeling assay with active casepase-3. The average numbers of venus fluorescence intensity in each cell, the average size of the venus punctum diameters, and caspase-3 fluorescence intensity were analyzed. **b** V1S and SV2 cells were co-cultured in the presence or absence of pam3CSK4 (10 μg/ml) (n = 3). **c** V1S and SV2 cells were co-cultured with either LV-control or LV-TLR2 (n = 3). **d** V1S and SV2 cells were co-cultured with either LV-sh.control or LV-shTLR2 (n = 3). **e** V1S and SV2 cells were co-cultured with either IgG (5 μg/ml) or T2.5 (5 μg/ml) (n = 3). **f** The kinetics of α-synuclein internalization in the presence of antibodies. dSY5Y cells were incubated with αSCM for indicated hours in the presence of either IgG (5 μg/ml) or T2.5 (5 μg/ml). The kinetics was analyzed by immunolabeling assay (n = 3). **g** Neuronal internalization of α-synuclein in the presence of antibodies. Mouse primary cortical neurons were incubated with αSCM or LZCM for indicated hours in the presence of either IgG (5 μg/ml) or T2.5 (5 μg/ml). Neurons were double immunolabelled with human α-synuclein (Middle panels) and active form of caspase-3 (low panels) (n = 3). Data are mean ± SEM (n = 3 per group). *p < 0.05, **p < 0.01, and ***p < 0.001; unpaired t test for all analysis except (**g**) (one way ANOVA). Scale bar, 20 μm

In this study, we showed that α-synuclein neuropathology was modulated by TLR2 in the α-Syn-tg model and the alterations rescued by an anti-TLR2 antibody. The α-synuclein pathology was significantly increased by TLR2 overexpression, however, decreased by its functional inhibition. Therefore, we proposed two potential mechanisms as to how TLR2 modulate α-synuclein pathology. First, neuronal TLR2 might modulates pathological α-synuclein accumulation through intra-neuronal signaling. Second, TLR2 may mediate neuron-to-neuron and neuron-to-glial α-synuclein propagation. We previously demonstrated that neuronal TLR2 modulated α-synuclein through autophagy inhibition [8]. Therefore, herein, we examined the roles of neuronal TLR2 in the pathological neuron-to-neuron α-synuclein propagation (Fig. 6). To verify our hypothesis, we first modulated the activity and expression of TLR2 in in vitro α-synuclein transmission live cell monitoring system that we developed and refer to as the dual-cell bimolecular fluorescence complementation (BiFC) system (Fig. 6a; Additional file 1: Figure S3a) [29, 39]. The system consists of neuronal donor cells (V1S) and neuronal recipient cells (SV2). V1S cells are expressing α-synuclein conjugated with amino-terminal fragment of venus (VN-α-syn) and SV2 cells are expressing α-synuclein conjugated with carboxy-terminal fragment of venus conjugated α-synuclein (α-syn-VC). Upon combining, the two proteins form the complete Venus fluorescence molecule (Additional file 1: Figure S3a). We previously have shown that this venus puncta is also human α-synuclein, phosphor-α-synuclein, and ubiquitin positive [29]. In addition, venus complementation did not observed when the V1S and SV2 cells were co-cultured with N-terminal (V1) or C-terminal venus only expressing (V2) cells [29]. Cell-to-cell transmission and the resulting co-aggregation between the transferred and endogenous α-synuclein proteins can be visualized and quantitatively analyzed by monitoring the Venus fluorescence (Additional file 1: Figure S3a). To avoid physical contacts between donor and recipient cells, the V1S and the SV2 cells were incubated in trans-well inserts and in lower compartments, respectively separated by a 0.4 μm pore membrane (Additional file 1: Figure S3b). In support of the validity of this system, the levels of Venus fluorescence in the recipient cells increased in proportion to the duration of co-culture, while fluorescence was not detected in single cell cultures (Additional file 1: Figure S3c). To examine the role of TLR2 in neuron-to-neuron α-synuclein transmission, we activated TLR2 using a conventional agonist, pam3CSK4 (Fig. 6b). Treatment with pam3CSK4 significantly increased both the fluorescence levels and the diameter of fluorescent puncta in recipient neuronal cells (Fig. 6b). TLR2 activation also increased the activity of caspase-3 in recipient cells (Fig. 6b). Similarly, overexpression of TLR2 with lentiviral vectors significantly increased the fluorescence levels, the diameter of fluorescent puncta, and caspase-3 activity in recipient cells (Fig. 6c). In contrast, these increases were reversed by lentivirus-mediated TLR2 gene knockdown (Fig. 6d) and anti-TLR2 treatment (Fig. 6e). Since we demonstrated TLR2 mediated neuron-to-neuron α-synuclein transmission, we next examined the role of TLR2 in α-synuclein internalization process by recipient neurons (Fig. 6f). Differentiated SH-SY5Y neuronal cells (dSY5Y) were exposed to α-synuclein conditioned medium (αSCM) [7] which contains neuron-released α-synuclein for 8 and 24 h α-synuclein (Fig. 6f). In neuronal recipient cells, exposure of αSCM led to internalization of α-synuclein in a time-dependent manner (Fig. 6f). At 8 h, internalized α-synuclein formed small intracellular puncta, but formed large inclusion body-like aggregates at 24 h (Fig. 6f). However, it was significantly decreased when dSY5Y cells were exposed to αSCM in the presence of T2.5 (Fig. 6f). Similarly, treatment with the αSCM resulted in increased internalization of α-synuclein and activation of caspase-3 in primary neurons, while treatment with the ant-TLR2 antibody significantly blocked internalization of α-synuclein and activation of caspase-3 (Fig. 6g). Human α-synuclein was not detected and caspase-3 activity was not affected in mouse primary neurons treated with LZCM (control conditioned medium, obtained from β-galactosidase overexpressing neuronal cells) (Fig. 6g). Collectively, these results support that TLR2 modulates neurotoxic α-synuclein accumulation through mediation of neuron-to-neuron α-synuclein transmission and that treatment with the neutralizing TLR2 antibody reduces α-synuclein accumulation and neurotoxicity.

### Antagonizing TLR2 decreases astroglial α-synuclein accumulation and inflammatory responses

**Fig. 7:**
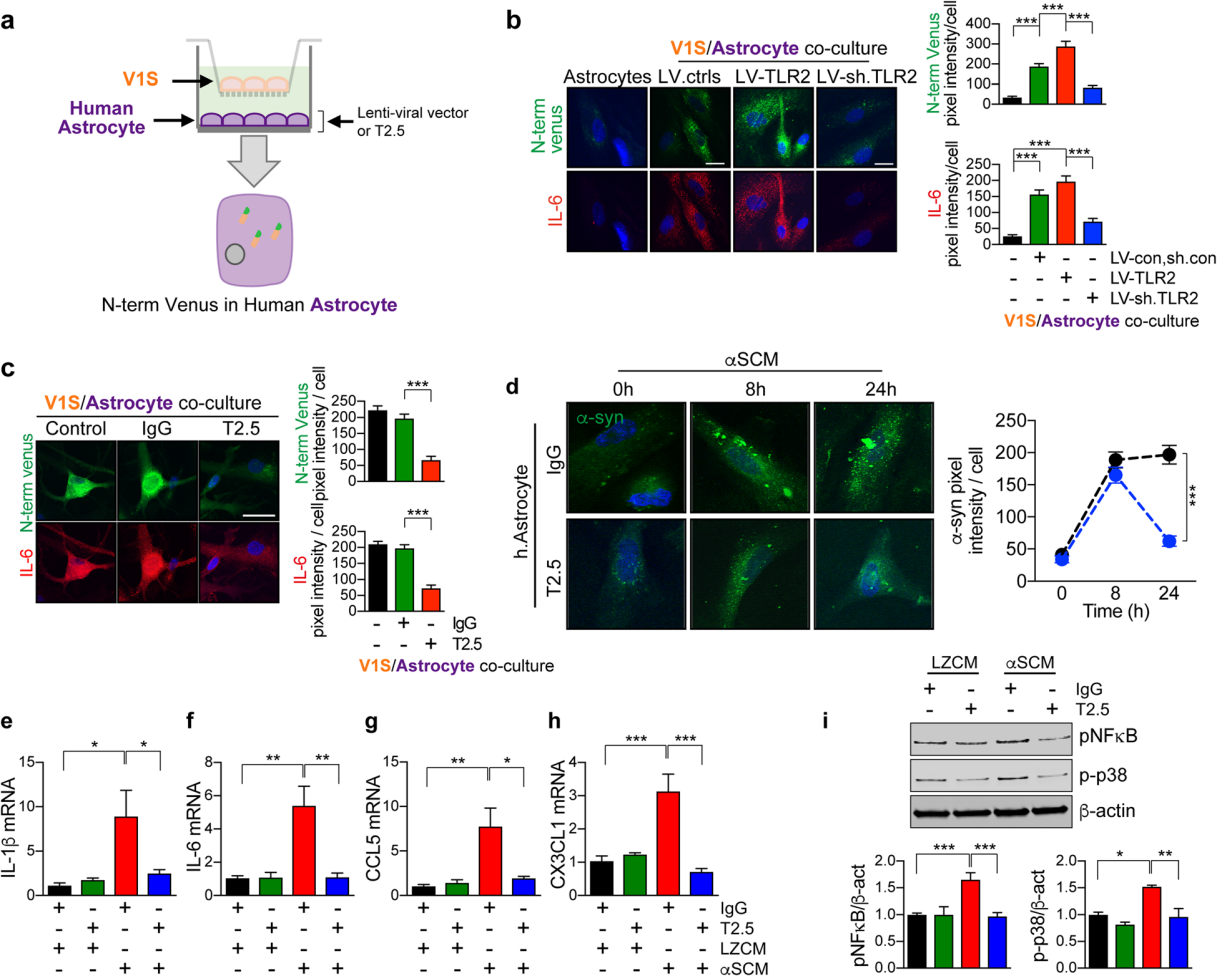
Astrocyte responses by TLR2 mediated neuron-to-astrocyte α-synuclein transmission. **a** Overview diagram. Donor neuronal cells (V1S), expressing α-synuclein-conjugated with N-terminus of venus were plated in trans-well insert and the recipient human primary astrocytes were plated onto the cover slips in the bottom well. Only astrocytes were treated with either lentiviral vectors or antibodies. Images were taken from astrocytes after a 3-days co-culture. **b**, **c** Representative confocal images for N-terminus of venus (Upper panel) and IL-6 (Lower panel) in recipient astrocytes. The fluorescence intensity of N-term venus and IL-6 were analyzed in randomly chosen area. **b** V1S and astrocytes were co-cultured in the presence of either LV-control/sh.control, LV-TLR2, or LV-sh.TLR2 (n = 3). **c** V1S and astrocytes were co-cultured in the presence of either IgG (5 μg/ml) or T2.5 (5 μg/ml) (n = 3). **d** The kinetics of astroglial α-synuclein internalization in the presence of antibodies. Human primary astrocytes were incubated with αSCM for indicated hours in the presence of either IgG (5 μg/ml) or T2.5 (5 μg/ml). The kinetics was analyzed by immunolabeling assay (n = 3). **e–h** Quantitative analysis of the cytokine/chemokine gene expressions in astrocytes. The cells were incubated with either LZCN or αSCM for 24 h in the presence of indicated antibodies. The expressions of IL-1β (**e**), IL-6 (**f**), CCL5 (**g**), and CX3CL1 (**h**) were normalized to the levels of β-actin (n = 4). Data are mean ± SEM. *p < 0.05, **p < 0.01, and ***p < 0.001; one way ANOVA for all analysis except (**d**) (unpaired t test). Scale bar, 20 μm

Since the astrocytes in the α-Syn-tg mice do not express human-α-synuclein and TLR2 expression is increased in glial cells in these mice (Fig. 1e and f), our findings indicate that the neuro-inflammation and increased accumulation of α-synuclein in glial cells in the α-Syn-tg (Fig. 4), might be the result of neuron to astrocyte transmission. Moreover, since T2.5 ameliorated these effects (Fig. 4) we propose that TLR2 might be a mediator of the accumulation of α-synuclein in glial cells. To better understand the mechanisms of action of the antibody, we utilized a modified in vitro α-synuclein transmission live cell (neuron-to-astrocyte) monitoring system (Fig. 7a). We co-cultured donor neurons with recipient human primary astrocytes (Fig. 7a). Donor neuronal cells (V1S), expressing the amino-terminal venus-α-synuclein fusion protein, were incubated in trans-well inserts and recipient astrocytes were placed in lower compartments (Fig. 7a). After 3 days of co-culture, the recipient astrocytes were immune-labelled with anti-venus antibody to verify the transferred N-term venus-tagged α-synuclein from neuronal V1S cells (Fig. 7a). Immunolabeling analysis revealed the transmission of venus-tagged α-synuclein from neuronal cells to astrocytes after a 3-day co-culture (Fig. 7b). In addition, overexpression of TLR2 significantly increased the immune-reactivity against venus protein in astrocytes, while it was decreased by lentiviral vector-mediated TLR2 gene knockdown and anti-TLR2 administration (Fig. 7b and c). Co-culture of astrocytes with α-synuclein-expressing neuronal cells also induced expression of IL-6, a pro-inflammatory cytokine gene, in astrocytes (Fig. 7b and c). Although TLR2 overexpression did not induce further increase of IL-6 expression in co-cultured astrocyte, IL-6 levels were significantly reduced by lentiviral vector-mediated TLR2 gene knockdown or by treatment with the anti-TLR2 antibody (Fig. 7b and c).

Next, we examined the role of TLR2 in α-synuclein internalization in recipient astrocytes and if treatment of anti-TLR2 blocks this process (Fig. 7d). Primary human astrocytes were exposed to αSCM for 8 and 24 h in the presence of either control IgG or anti-TLR2 (Fig. 7d). In recipient astrocytes, internalization of α-synuclein was increased in both IgG and anti-TLR2 treated cells at the early time point (8 h) (Fig. 7d). However, it was significantly reduced by TLR2 inhibition at the late time point (24 h) (Fig. 7d). This is consistent with the in vivo studies showing increased accumulation of α-synuclein in astrocytes in the IgG treated α-Syn-tg and that anti-TLR2 treatment reduced the astroglial accumulation of α-synuclein (Fig. 4c). Moreover, exposure to extracellular α-synuclein induced astroglial expressions of the pro-inflammatory cytokines and chemoattractant chemokines, such as IL-1β, IL-6, CCL5, and CX3CL1, while those elevations were completely inhibited by anti-TLR2 treatment (Fig. 7e-h). Immunoblotting analysis also revealed that exposure to α-synuclein induced activation of NFκB and p38 MAPK in astrocytes (Fig. 7i). Collectively, these results suggest that TLR2 modulates astroglial α-synuclein accumulation through neuron-to-astrocyte α-synuclein transmission, thereby regulates astroglial responses, and the blocking of this effect might underlie the neuroprotective and immunomodulatory effects of the anti-TLR2 treatment.

## Discussion

The present study showed that administration of anti-TLR2 alleviated α-synuclein accumulation in neuronal and astroglial cells, neuroinflammation, neurodegeneration, and functional deficits in the mouse model of PD/DLB. Moreover, in vitro studies with neuronal and astroglial cells showed that the anti-TLR2 blocks NFκB dependent pro-inflammatory responses by blocking the neuron-to-neuron and neuron-to-astrocyte α-synuclein transmission. While for this study we focused on the effects of blocking TLR2 on glial and NFκB dependent neuro-inflammatory responses, in previous studies we investigated the pathological roles of TLR2 in neurons in in vitro and in vivo models of synucleinopathy [8]. In neurons, extracellular α-synuclein inhibited autophagy in a TLR2 dependent manner via mTOR and AKT signaling cascades [8]. Thus, activation and gene overexpression of TLR2 induced abnormal accumulation of α-synuclein aggregates in neuron followed by accumulation autophagy markers, such as p62SQS/TM1 [8]. We have also shown the pathogenic interaction of TLR2 and α-synuclein in microglia [7]. The oligomeric forms of extracellular α-synuclein interacts with TLR2 on the surface of microglia, thereby induced neurotoxic microglia activation through NFκB and p38 MAPK signaling cascades [7]. Once activated, microglia produced neurotoxic by-products, such as inflammatory cytokines, reactive oxygen species, and nitric oxides [7].

**Fig. 8:**
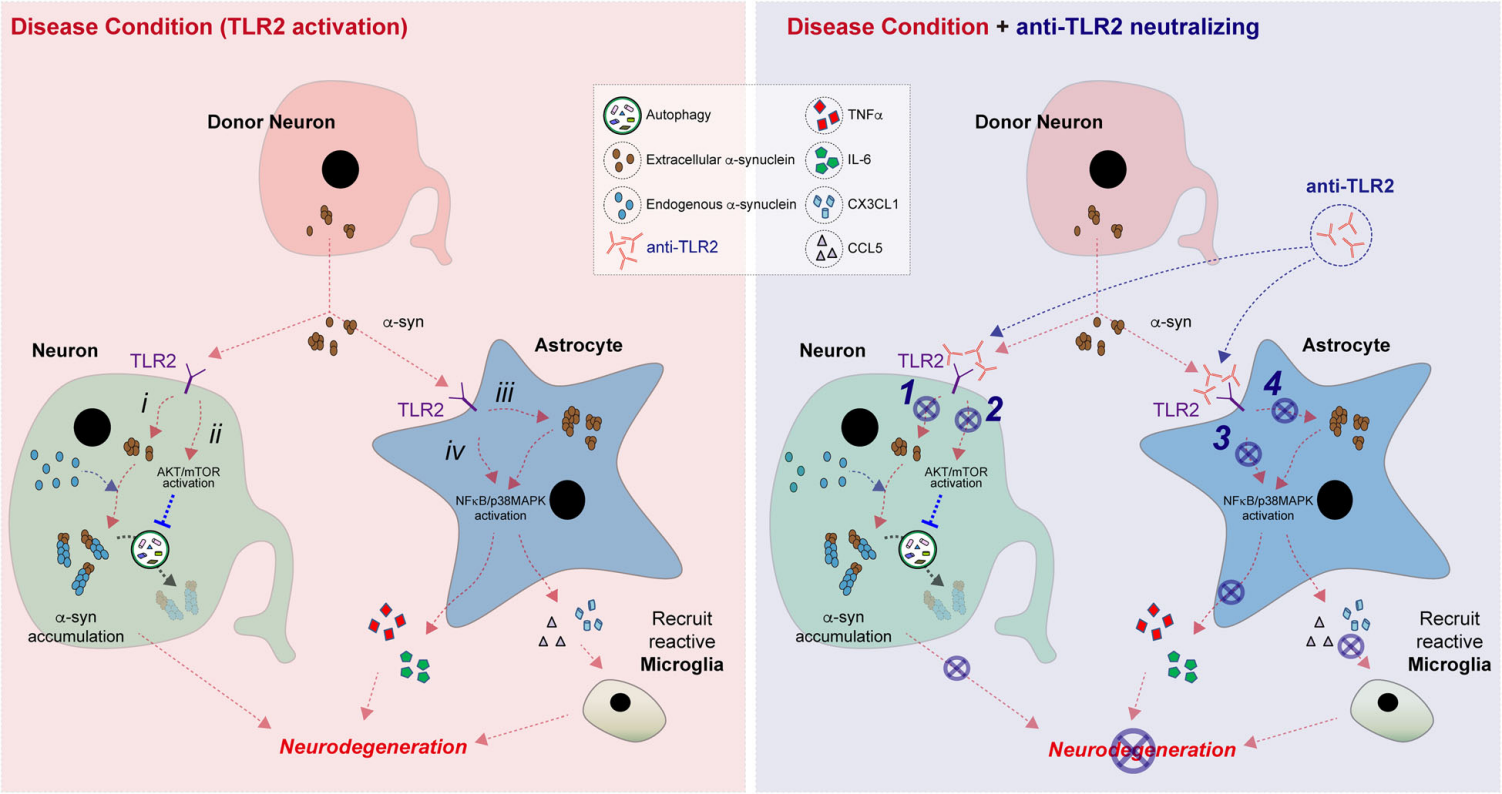
Model for TLR2 immunotherapy ameliorates neurodegeneration in synucleinopathy. In disease condition, TLR2 mediates neurotoxicity. In neuron, i) TLR2 induces pathological internalization of extracellular α-synuclein into neuron and ii) extracellular α-synuclein activates neuronal TLR2 which results in mTOR-mediated autophagy inhibition. Thus, neuronal TLR2 induces neurotoxic α-synuclein accumulation. In astrocyte, iii) TLR2 increased abnormal α-synuclein accumulation which leads astroglial activation and iv) extracellular α-synuclein activates astroglial TLR2 signaling cascade through NFκB/p38 MAPK which results in neurotoxic astroglial responses such as pro-inflammatory cytokine expression and induction of reactive microglia recruiting chemokines. Therefore, TLR2 immunotherapy ameliorates α-synuclein-mediated neurotoxicity via inhibition of 1) TLR2-mediated neuronal α-synuclein internalization, 2) activation of neuronal autophagy via TLR2-mTOR signaling cascade, 3) inhibition of TLR2-mediated astroglial responses, and 4) reduction of astroglial α-synuclein accumulation. Thereby, TLR2 immunotherapy might be a novel therapeutic strategy for synucleinopathy

Another new finding of this study, is that in addition of the microglia, extracellular α-synuclein induced astroglial responses which are neurotoxic. Once exposed to α-synuclein, astrocytes expressed pro-inflammatory cytokine expressions through NFκB and p38 MAPK signaling cascades (Fig. 8). Therefore, we targeted TLR2 in in vivo and in in vitro models of synucleinopathy using a functional inhibitory antibody (T2.5). Remarkably, the administration of TLR2 functional blocking antibody significantly reduced α-synuclein depositions in neurons and astroglial cells as well ameliorating neurodegeneration, neuro-inflammation, and NFκB activation. The in vivo results were confirmed in vitro where treatment with the anti-TLR2 decreased internalization of α-synuclein into neuron and astrocytes and this was accompanied by decreased expression of pro-inflammatory cytokines and signaling via the p38/MAPK and NFκB pathway. Therefore, these results support that TLR2 plays key roles in the pathogenesis of synucleinopathy and functional inhibition of TLR2 ameliorates neuropathology in synucleinopathy through inhibition of pathogenic neuron-to-neuron and neuron-to-astrocyte α-synuclein transmission, clearance of accumulated neurotoxic α-synuclein via autophagy, and inhibition of astroglial inflammatory responses (Fig. 8).

The steady state levels of α-synuclein could be affected by various different factors including gene expression and proteostasis. In particular, it has been suggested that the clearance of α-synuclein could be determined by assembly state of the protein [4, 40]. Autophagy/lysosomal pathway is involved in the clearance of oligomeric and fibrilar species of α-synuclein [41–43], while monomeric and dimeric α-synuclein species are degraded by ubiquitin-proteasome system and chaperone-mediated autophagy [44, 45]. We previously have shown that functional inhibition of TLR2 activated autophagy process in neuron [8]. Consistent with these findings, in current study, we observed a significant reduction of triton-insoluble α-synuclein oligomers in T2.5 administrated α-Syn-tg while the level of α-synuclein monomer was not affected by antibody administrations (Fig. 3d). Together, these results suggest that administration of TLR2 functional inhibitory antibody reduced accumulation of neurotoxic α-synuclein oligomers through autophagy-mediated clearance in an animal model of PD.

There is growing evidence that receptor-mediated transmission of α-synuclein is responsible for the spreading of synucleinopathy lesions in PD/DLB. Recent studies have suggested LAG3 that might be such a receptor that operates by mediating seeding and transmission of α-synuclein fibrils [9], but the details are still largely unknown. Our study is different in that we focused on the effects of blocking TLR2 which is a mediator of the neurotoxic and pro-inflammatory of extracellular α-synuclein oligomers. We have previously shown that TLR2 is not activated by recombinant fibrils as is the case for LAG3 but rather by neuron-released extracellular α-synuclein oligomers [7]. Using α-synuclein conformation specific antibodies, we also demonstrated that α-synuclein oligomers mainly contributed to neuron-to-neuron α-synuclein transmission/propagation instead of fibril forms in a recent study [46]. Neuron-to-neuron α-synuclein transmission was significantly decreased in the presence of oligomer conformation specific antibodies in this BiFC α-synuclein monitoring system, while it was not affected by fibril specific antibodies [46]. In addition, the caspase-3 activities were increased in proportion to the levels of small size of venus puncta in recipient neuronal cells. Together, these results support that oligomer is a main contributor of pathogenic cell-to-cell α-synuclein transmission.

Although most studies have focused at investigating the accumulation of α-synuclein in neurons, there is growing evidence that α-synuclein accumulates in astrocytes in the brains of patients with PD/DLB [47–51]. Consistent with these observations, in the present study we show that α-synuclein accumulates in astrocytes of the α-Syn-tg mice and that is associated with increased TLR2 expression, pro-inflammatory cytokines, and NFκB activation. Treatment with anti-TLR2 blocked these effects in in vivo. We have previously shown that neuron-to-astrocyte α-synuclein transmission might be an undelaying pathway for α-synuclein accumulation in astrocytes [33]. In addition, in the current study, we verified the central role of TLR2 in astroglial α-synuclein accumulation. Overexpression of astroglial TLR2 significantly increased astroglial α-synuclein internalization as well as neuron-to-astrocyte α-synuclein transmission in in vitro synucleinopathy model system, while those were inhibited by anti-TLR2 treatment and TLR2 gene knockdown. Interestingly, the internalization of α-synuclein was not affected by TLR2 functional inhibition at the early time point in α-synuclein-exposed astrocyte. This result suggests the existence of TLR2-independent astrocyte-specific α-synuclein internalization mechanisms. However, the level of internalized α-synuclein was significantly reduced by TLR2 functional inhibition at the late time point exposure. This also suggests that TLR2 activity might be associated with a clearance mechanism of accumulated α-synuclein in the astrocyte. In addition, TLR2-dependent astroglial α-synuclein accumulation triggered neurotoxic astroglial pro-inflammatory responses through NFκB and p38 MAPK signaling cascades (Fig. 8). Interestingly, α-synuclein-exposed astrocytes also induced chemoattractant chemokine expressions, such as CCL5 and CXCL1 which are recruiting neurotoxic reactive microglial cells into affected brain regions [52]. Thereby, these findings suggest that TLR2 mediates astroglial α-synuclein accumulation through neuron-to-astroglial α-synuclein transmission and may contribute to local immune response in patients with DLB/PD, and that treatment with anti-TLR2 antibody might block the neuropathology by blocking NFκB and p38 MAPK signaling in astrocytes.

While the numbers of TLR2 positive microglia cells were increased in the brains of PD/DLB patients and mouse models [7, 34, 53], we were not able to observed extensive microglial α-synuclein deposition in our current in vivo study. In addition, we failed to demonstrate the neuron-to-microglial α-synuclein transmission using the in vitro transmission assay. Instead, we found that once internalized, α-synuclein was rapidly cleared from microglia regardless of TLR2 activity (data not shown). Together with our previous findings [54], these results suggest that microglia are the most efficient cells for removal of extracellular α-synuclein in the brain and may have a distinct specific mechanism for degradation and clearance of internalized α-synuclein aggregates. Therefore, microglia might be a candidate for cell-based therapeutics against synucleinopathies to remove pathogenic forms of extracellular α-synuclein.

For over two decades, immunotherapy has been proposed as a potential treatment approach for neurodegenerative disorders of the aging population such as AD and PD/DLB. In the field of synucleinopathies, considerable progress has been made by developing active [22, 55], passive [32, 46, 56–60], and cellular [19, 61, 62] immunotherapeutic approaches of which a few of them have moved to clinical trials [63]. Since α-synuclein is a key pathological mediator in the disorders with parkinsonism and dementia, most of studies have targeted monomeric, oligomeric, and fibrilar α-synuclein [64]. However, the characteristics of the physiological vs pathogenic forms of α-synuclein is still enigmatic [65]. Thus, in addition of targeting α-synuclein it might necessary to develop therapeutics for alternative pathways such as neuro-inflammation, autophagy, transcriptional regulation, and mitochondrial energetic and biogenesis [4, 65].

## Conclusions

In this context we propose immunotherapy targeting TLR2, given its role as a mediator of the neurotoxic and pro-inflammatory effects of extracellular α-synuclein oligomers. Such therapy might be suitable for combinatorial approaches with molecules interfering with α-synuclein or pathways relevant to neuro-inflammation, autophagy and mitochondrial energetic and biogenesis [27]. In summary, we propose that TLR2 is a novel target for immunotherapy and a potentially viable therapeutic strategy for synucleinopathies of the aging population.

## Additional file

Additional file 1: **Figure S1**, related to Fig. 2. Delivery of TLR2 overexpression lentiviral vectors into mouse model of synucleinopathy. **Figure S2**, related to Figs. 2 and 7. Human α-synuclein positive astrocytes in synucleinopathy mouse model. **Figure S3**, related to Figs. 6 and 7. Live α-synuclein cell-to-cell transmission monitoring system. (DOCX 3555 kb)

## Abbreviations

BiFC: Bimolecular fluorescence complementation
CCL5: Chemokine (C-C motif) ligand 5
CX3CL1: C-X3-C Motif Chemokine Ligand 1
DLB: Dementia with Lewy bodies
GFAP: Glial fibrillary acidic protein
IL: Interleukin
MAPK: Mitogen-activated protein kinase
NFκB: Nuclear factor kappa-light-chain-enhancer of activated B cells
PD: Parkinson’s disease
TH: Tyrosine hydroxylase
TLR2: Toll-like receptor 2
TNF: Tumor necrosis factor
