# Supplementary material for: Immunotherapy targeting toll-like receptor 2 alleviates neurodegeneration in models of synucleinopathy by modulating α-synuclein transmission and neuroinflammation
Source: Mol Neurodegener. 2018 Aug 9;13:43. doi: 10.1186/s13024-018-0276-2 (PMC6085656; doi:10.1186/s13024-018-0276-2)
Supplement: Supplementary file 1 — Figure S1, related to Fig. 2. Delivery of TLR2 overexpression lentiviral vectors into mouse model of synucleinopathy. Figure S2, related to Figs. 2 and 7. Human α-synuclein positive astrocytes in synucleinopathy mouse model. Figure S3, related to Figs. 6 and 7. Live α-synuclein cell-to-cell transmission monitoring system. (DOCX 3555 kb) [file 13024_2018_276_MOESM1_ESM.docx]

**Additional file 1**

**Supplemental Data**

**Figure S1, Related to Figure 2**

**
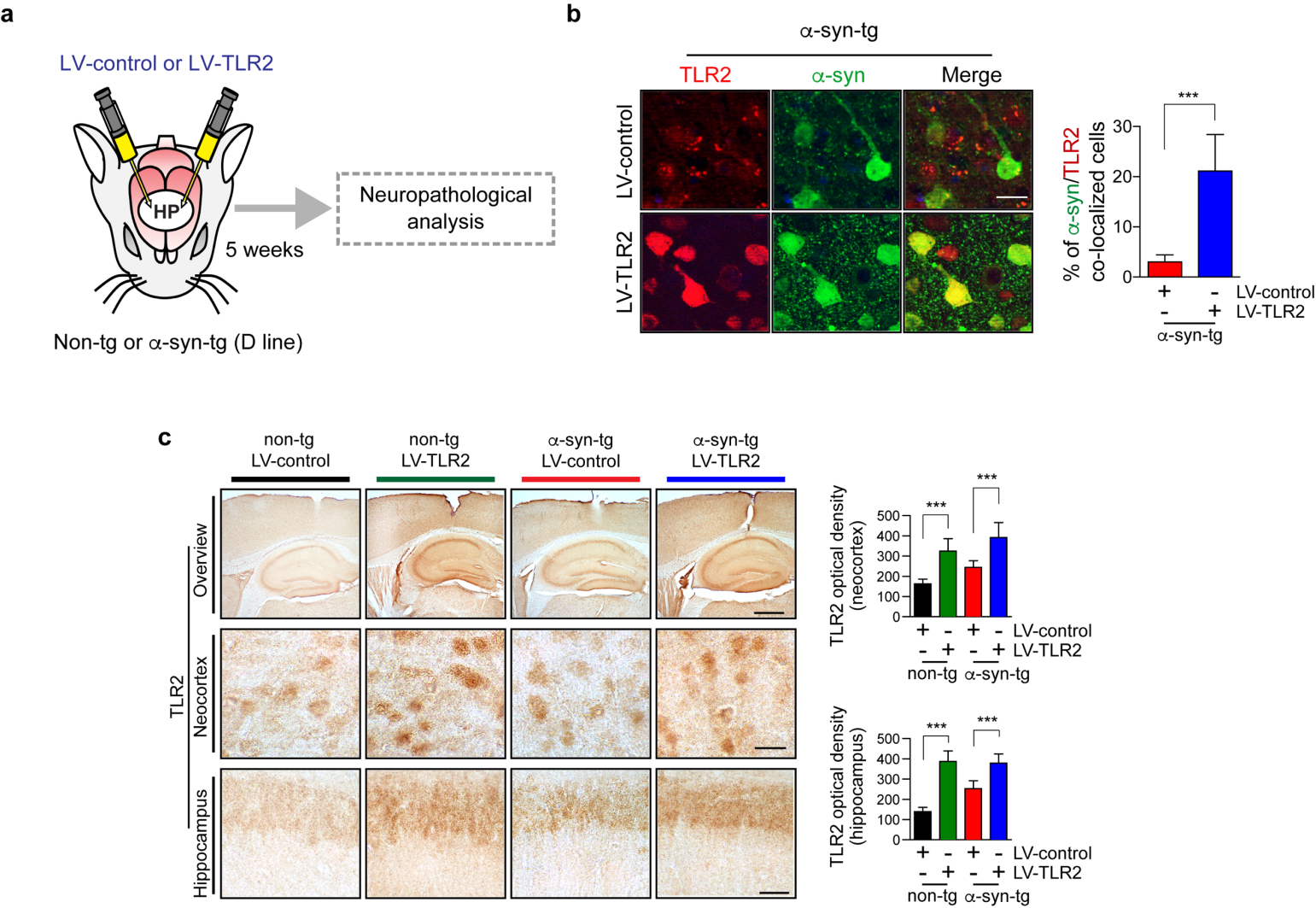
**

**Figure S1.** Delivery of TLR2 overexpression lentiviral vectors into mouse model of synucleinopathy. **a** Experimental scheme. Either LV-control or LV-TLR2 was injected into the hippocampus of non-tg or α-syn-tg mice (D line). Neuropathology was analyzed after a 5-weeks post injection. **b** Representative images from double immunostaining of TLR2 with α-synuclein in neocortex of lentiviral vector-injected α-syn-tg mice. The percentages of TLR2 and α-synuclein positive cells were analyzed in the neocortex of lentiviral vector injected α-syn-tg mice. (n = 6 per group; unpaired t test, ***p < 0.001). Data are represented as mean ± SEM. Scale bar, 10 μm. **c** Representative immunohistochemical staining of TLR2 in neocortex (middle panels) and hippocampus (low panels) of lentiviral vector-delivered mice. The level of TLR2 was analyzed in the neocortex and hippocampus of the mice by optical density quantification mice (n = 6 per group; one way ANOVA, ***p < 0.001). Data are represented as mean ± SEM. Scale bars, 100 μm (low magnification) and 20 μm (high magnification).

**Figure S2, Related to Figures 2 and 7**

**
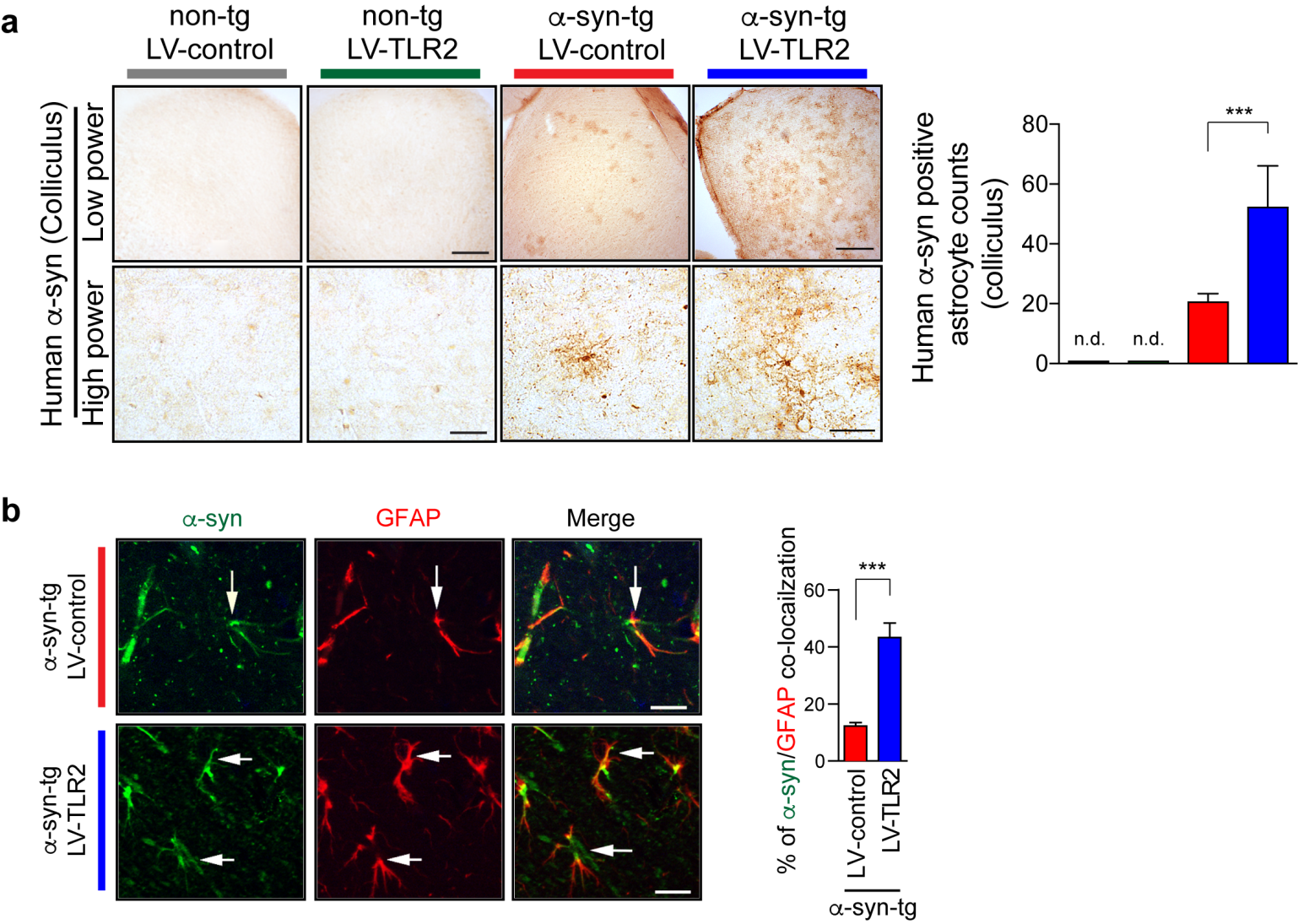
**

**Figure S2.** Human α-synuclein positive astrocytes in synucleinopathy mouse model. **a** Human α-synuclein positive astrocyte in colliculus of lentiviral vector delivered non-tg and α-syn-tg mice (D line). Representative images from immunohistochemical analysis of human α-synuclein in colliculus of non-tg and α-syn-tg mice. The numbers of human α-synuclein positive cells were counted in the colliculus of mice (n = 6 per group; unpaired t test, ***p < 0.001). Data are represented as mean ± SEM. Scale bars, 100 μm (low magnification) and 20 μm (high magnification). **b** Human α-synuclein positive astrocytes in the hippocampus of lentiviral vector delivered non-tg and α-syn-tg mice. Representative images from double-immunolabeling analysis for α-synuclein (green) and GFAP (red). The level of α-synuclein and GFAP-double positive cell was analyzed in α-syn-tg mice (n = 6 per group; unpaired t test, ***p < 0.001). Data are represented as mean ± SEM. Scale bars, 10 μm.

**Figure S3, Related to Figure 6 and 7**

**
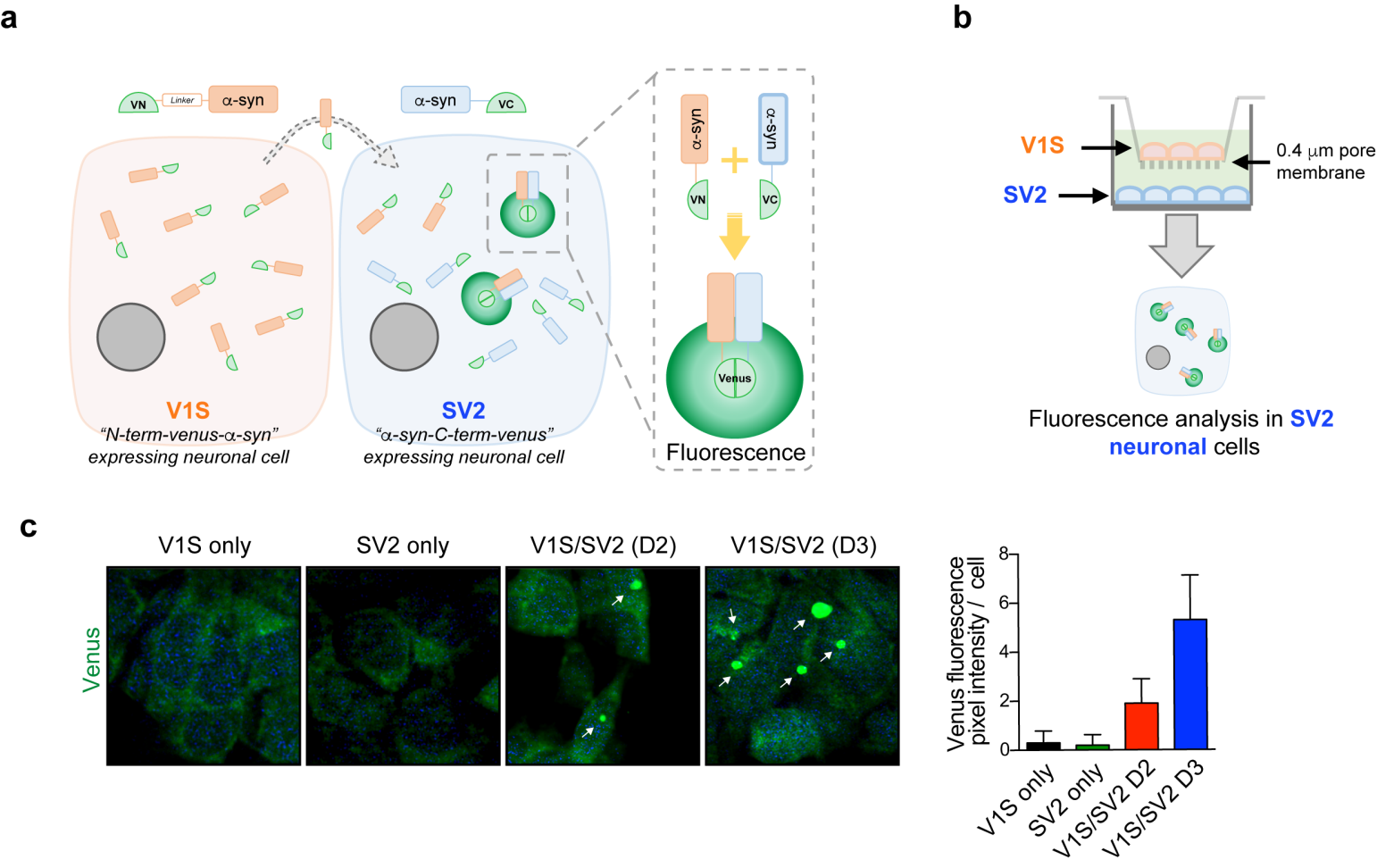
**

**Figure S3.** Live α-synuclein cell-to-cell transmission monitoring system. **a** Overview diagram of bimolecular fluorescence complementation (BiFC) based live α-synuclein cell-to-cell transmission monitoring system. Neuronal donor cells (V1S) are expressing “N-term-venus” conjugated α-synuclein (VN-α-syn) and recipient neuronal cells (SV2) are expressing α-synuclein conjugated with “C-term-venus” (α-syn-VC). Upon combining, the two proteins form the complete Venus fluorescence molecule. **b** The method for co-culture of donor V1S and recipient SV2 cells. V1S cells were placed in trans-well and SV2 cells were placed in the coverslips of low compartment to avoid physical contact. During the co-culture, VN-α-syn transferred from V1S to SV2 cell, thereby interacted with endogenous α-syn-VC, results in venus complementation (Bae et al., 2014). **c** Representative BiFC fluorescence images and quantification of venus florescence puncta of V1S only, SV2 only, V1S/SV2 co-cultured (for 2 days), and V1S/SV2 co-cultured (for 3 days). The signals observed V1S only and SV2 only are background. The venus signal was only detected in V1S/SV2 co-cultured cells (Arrows). The average number of venus fluorescence intensity in each cell was analyzed (n = 3).

**Supplemental reference**

Bae, E.J., Yang, N.Y., Song, M., Lee, C.S., Lee, J.S., Jung, B.C., Lee, H.J., Kim, S., Masliah, E., Sardi, S.P.*, et al.* (2014). Glucocerebrosidase depletion enhances cell-to-cell transmission of alpha-synuclein. Nat Commun *5*, 4755.
